# Supplementary material for: Potassium Iodide Doping for Vacancy Substitution and Dangling Bond Repair in InP Core-Shell Quantum Dots
Source: Nanomaterials (Basel). 2024 Jun 19;14(12):1055. doi: 10.3390/nano14121055 (PMC11206699; doi:10.3390/nano14121055)
Supplement: Supplementary file 1 [file nanomaterials-14-01055-s001.zip › nanomaterials-3024309-supplementary.pdf]

Supporting Information

Potassium Iodide Doping for Vacancy Substitution and Dangling Bond Repair in InP Core-Shell Quantum Dots

Ji-Eun Lee [1], Chang-Jin Lee [2], Seung-Jae Lee [2,3], Ui-Hyun Jeong [2] and Jea-Gun Park [1,2,]*

1 *Department of Information Display Engineering, Hanyang University, Seoul, 04763, Republic of Korea*

2 *Department of Electronic Engineering, Hanyang University, Seoul, 04763, Republic of Korea*

3 *Samsung Electronics, 130 Samsung-ro, Suwon, Gyeonggi-do 16678, Republic of Korea*

* *Correspondence: parkjgL@hanyang.ac.kr*

Experimental section

1. Materials

For the core and shell materials of QDs, Oleylamine (OLA, 70%, Sigma-Aldrich), trioctylphosphine (TOP, 97%, Sigma-Aldrich), diphenylphosphine (DPP, 98%, Sigma-Aldrich), paraffin oil (PO, viscose liquid, Sigma-Aldrich), 1-octadecene (ODE, technical 90%, Sigma-Aldrich), indium(III) acetate ($In(OAc)_3$, 99.99%, Sigma-Aldrich), zinc acetate ($Zn(OAc)_2$, 99.99%, Sigma-Aldrich), zinc stearate ($Zn(st)_2$, technical grade, Sigma-Aldrich), lauric acid (LA, >98%, Sigma-Aldrich), palmitic acid (PA, >99%, Sigma-Aldrich), oleic acid (OA, technical grade 90%, Sigma-Aldrich), stearic acid (SA, >95%, Sigma-Aldrich), potassium iodide (KI, 99%, Sigma-Aldrich), selenium (Se, 99.99% powder 100 mesh, Sigma-Aldrich), sulfur (trace metal basis 99.998%, Sigma-Aldrich), 1-dodecanethiol (DDT, >98%, Sigma-Aldrich), hydrofluoric acid (HF,48%, Sigma-Aldrich), Toluene (>99.9%, Daejung), hexane (>98.5%, Daejung), acetone (>99.8%, Daejung), ethanol (>99.5%, Daejung), n-octane (>97%, Daejung), and Tris(trimethylsilyl) phosphine ($(TMS)_3P$, 99.5%, SK Chemical) were employed.

2. Synthesis of core/multi-shell QDs

2.1 Preparation of stock solutions

For the synthesis of blue-light-emitting QDs, a 0.5 M solution of Se-DPP was prepared in a nitrogen (N_2)-filled glovebox by dissolving 5 mmol of Se powder and 1 mL of DPP in 9 mL of toluene. The 0.27 M $Zn(OA)_2$ precursor was prepared by dissolving 12 mmol of $Zn(OAc)_2$ and 24 mmol of OA in 32.5 mL of ODE, then the mixed solution was degassed at 150 °C for 1 h and heated up to 250 °C under a N_2 flow. In addition, for the synthesis of green and red QDs, a 0.316 M $Zn(st)_2$ precursor was prepared by dissolving 4.74 mmol of $Zn(st)_2$ in 15 mL of ODE, and the mixed solution was degassed and heated

using the same conditions. A 0.2 M solution of $(TMS)_3P$ was prepared in a N_2-filled glovebox by combining 2 mmol of $(TMS)_3P$ with 10 mL of TOP. A 1.79 M Se-TOP mixed solution was prepared in a N_2-filled glovebox by dissolving 17.9 mmol of Se powder in 10 mL of TOP. A 0.1M HF-acetone mixed solution was prepared by dissolving 1.4 mmol of HF in 14 mL of acetone. After that, all mixed solutions were degassed at 200 °C for 30 min in a N_2-filled glovebox.

2.2 Synthesis of blue-light-emitting ZnSe/ZnS core/shell QDs

For the ZnSe core, 1 mmol of $Zn(OAc)_2$ and 2 mmol of OA were loaded into a 100-mL 3-neck flask with 12 mL PO at room temperature (RT). The mixed solution in flask was heated up to 150 ˚C with stirring, followed by degassing under a pressure of 100 mTorr for 60 min. Subsequently, the distilled mixed solution in flask was heated up to 360 ˚C under N_2 flow to obtain a colorless transparent solution of $Zn(OA)_2$. And then, a Se-DPP stock solution in 9 mL of toluene was rapidly injected into mixed solution in flask at 360 ˚C. After the injection of Se-DPP stock solution, the ZnSe core was grown at the same temperature for 35 min. Next, 4 mL of a 0.27 M $Zn(OA)_2$ precursor was additionally injected and the mixed solution in flask was kept for 5 min. In addition, 1 mL of a 0.5 mM Se-DPP precursor in 9 mL of toluene was injected into the flask and kept at the same temperature for 15 min. For the second-layer of ZnSe, the mixed solution in flask was coated with the 4 mL of 0.27 M $Zn(OA)_2$ precursor and 1 mL of 0.5 mM Se-DPP precursor by using the same as previous process. To grow the ZnS shell on the synthesized ZnSe core, the mixed solution in flask of the ZnSe core was cooled down to 330 °C without any pre-purification process of the core solution, and 4.8 mL of a 0.27 M $Zn(OA)_2$ precursor was injected and kept for 30 min at this temperature. Then, 0.48 mL of the DDT was injected into the flask. After 30 min, to obtain blue-light-emitting ZnSe/ZnS QDs, the mixed solution in flask was cooled down to RT and then centrifuged three times with acetone and ethanol to eliminate the impurities generated from

unreacted precursors and byproducts. Finally, the precipitated blue-light-emitting QDs were redispersed in hexane and it stored at RT for the application of QD-functional CF-OLED hybrid display. A schematic synthesis process was shown in **Figure S2.**

2.3 Synthesis of green-light-emitting InP core QDs

For the InP core, 0.42 mmol of $In(OAc)_3$ and 1.26 mmol of LA were loaded into a 100-mL 3-neck flask with 6 mL of ODE at RT. The mixed solution was stirred and heated up to 150 ˚C, then degassed under a pressure of 100 mTorr for 60 min. Subsequently, 5.3 mL of a 0.27M $Zn(OA)_2$ solution was rapidly injected into the mixed solution in flask at 150 ˚C under a flow of N_2. The distilled mixed solution in flask was then heated up to 240 ˚C to obtain a colorless transparent solution of $In(LA)_3$ and $Zn(OA)_2$. A precursor solution of 0.68 mL of 0.2mM $(TMS)_3P$ was rapidly injected into the mixed solution in flask at 240 ˚C. And then, the InP core were grown for 10 min at that temperature. Afterward, to obtain green-light-emitting InP core QDs, the mixed solution in flask was cooled down to RT and then centrifuged twice with acetone to eliminate the impurities generated from unreacted precursors and byproducts. Finally, the prepared QDs were redispersed in 5 mL of toluene.

2.4 Synthesis of green-light-emitting InP/ZnSe/ZnSeS/ZnS core/multi-shell QDs

To grow the ZnSe shell on the synthesized InP core, a 100-mL 3-neck flask was prepared by mixing 2 mL of 0.316 M $Zn(st)_2$ with 15 mL of ODE. The mixed solution was stirred and heated up to 150 ˚C, then degassed under a pressure of 100 mTorr for 60 min. Subsequently, the InP core in 5 mL of toluene was injected into the mixed solution in flask at 150 °C and treated with 1.4mL of HF-acetone under a flow of N_2. The mixed solution was heated up to 180 °C, and 0.24 mL of 1.79 M Se-TOP precursor was injected and kept at 180 °C for 10 min. And then, the mixed solution in syringe of 2.3 mL of 0.316M $Zn(st)_2$ precursor was injected dropwise into the mixed solution in flask, the

mixed solution in flask was kept at 200 °C for 10 min. After that, 0.21 mL of 1.79M Se-TOP precursor was injected into the mixed solution in syringe, the mixed solution in flask was kept at 220 °C for 10 min. In addition, the mixed solution in syringe of 2.9 mL of 0.316M $Zn(st)_2$ precursor was injected dropwise into the mixed solution in flask, the flask was kept at 240 °C for 10 min. After injecting a 0.2 mL mixed solution in syringe of 1.79 M Se-TOP precursor, the mixture solution in flask was kept at 260 °C for 10 min. For the passivation of ZnSe/ZnSeS multi-shell, after the mixed solution in syringe of 0.9 mL of 0.316 M $Zn(st)_2$ precursor was injected dropwise, the mixed solution in flask was kept at 260 °C for 20 min. Then, after the mixed solution in syringe of 0.28 mL of $Se_{0.3}S_{0.7}$-TOP precursor was injected dropwise, the mixed solution in flask was kept at 300 °C for 20 min. In addition, the mixed solution in syringe of 0.9 mL of 0.316 M $Zn(st)_2$ precursor was injected dropwise, the mixture in flask was kept at 320 °C for 20 min. After that, the mixed solution in syringe of 0.28 mL of $Se_{0.15}S_{0.85}$-TOP precursor was injected, the mixed solution in flask was kept at 320 °C for 40 min. For the passivation of ZnS outer-shell, the mixed solution of InP/ZnSe/ZnSeS core/multi-shell was cooled down to 230 °C, and at this temperature, 6 mL of 0.27 M $Zn(OA)_2$ precursor was injected and kept for 30 min. Next, 0.64 mL of DDT was injected into the mixed solution in flask, and the temperature was kept. To obtain green-light-emitting InP/ZnSe/ZnSeS/ZnS core/multi-shell QDs, the mixed solution in flask was cooled down to RT after 30 min. and then centrifuged three times with acetone and ethanol to eliminate impurities from unreacted precursors and byproducts. Finally, the precipitated QDs were redispersed in hexane and it stored at RT for application of QD-functional CF-OLED hybrid display applications. A schematic synthesis process was shown in **Figure S3.**

Table S1. Summary of optical properties of undoped and KI-doped red-light-emitting InP-based core/shell QD.

KI doping concentration [%]	PL λ_{max}[a] [nm]	FWHM [nm]	Abs. λ_{max}[b] [nm]	Stokes shift[c] [nm]	PL-QY[d] [%]	τ_{avg} [ns]
Undoping	617	43	597	21	74	43
1% KI	622	42	599	23	83	46
3% KI	622	40	599	23	97	49
5% KI	628	43	601	28	88	45
7% KI	631	44	601	30	79	45

[a] Wavelength of photoluminescence peak. [b] First peak wavelength of absorption spectra. [c] The difference (in wavelength) between abs. λmax and PL λmax. [d] Measured by absolute PL-QY using a QE-2100(Otsuka Electronics). [e] Measured by exciton lifetime using a TRPL(Time-resolved photoluminescence).

Table S2. Components of biexponential decay fitting for TRPL measurements of InP-based core/multi-shell QDs with varying KI doping concentrations [S1].

Sample	A_1	τ_1 [ns]	A_2	τ_2 [ns]	τ_{avg} [ns]	τ_1% [ns]	τ_2% [ns]
**Un doped**	0.53	27	0.25	59	43	49	51
**1 % KI**	0.54	28	0.24	64	46	50	50
**3 % KI**	0.57	31	0.25	67	49	51	49
**5 % KI**	0.56	30	0.25	62	45	50	50
**7 % KI**	0.59	29	0.28	61	45	50	50

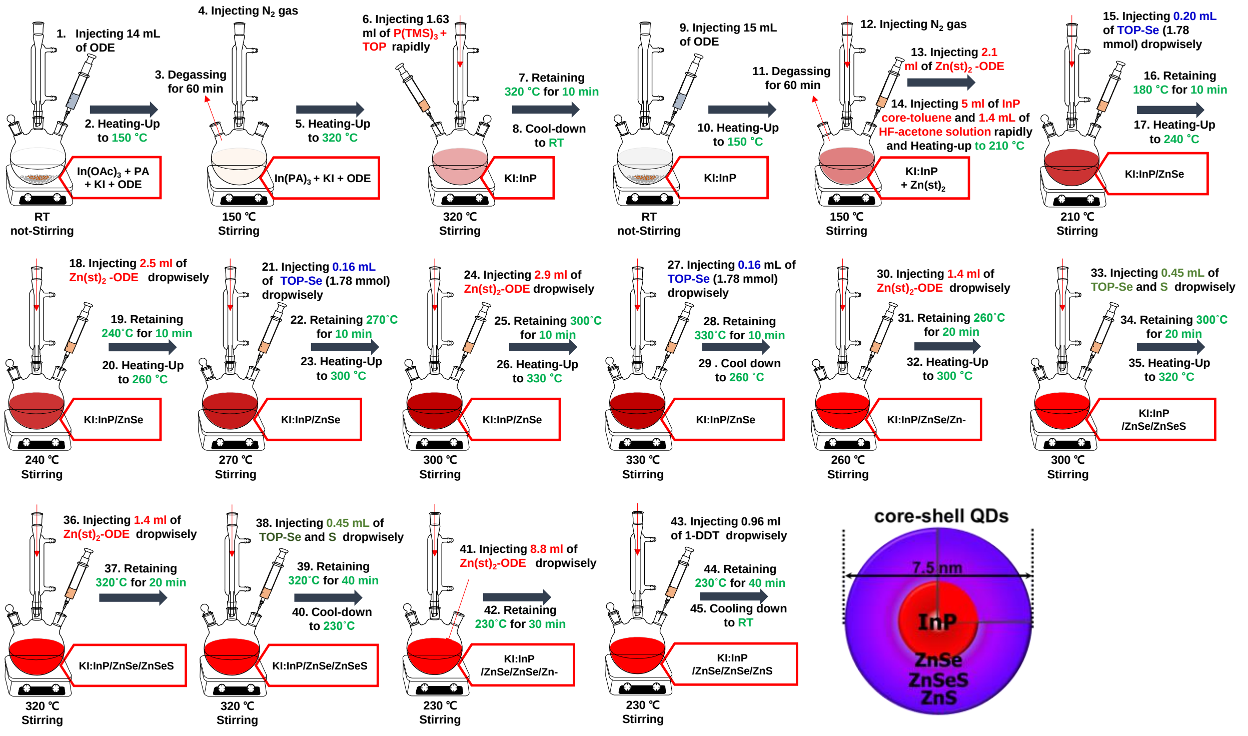

Figure S1. Schematic diagram for the synthesis process of red-light-emitting KI-doped $In_{0.53}P_{0.47}/Zn_{0.6}Se_{0.4}/ Zn_{0.6}Se_{0.1}S_{0.3}/Zn_{0.5}S_{0.5}$ core/ multi-shell QDs.

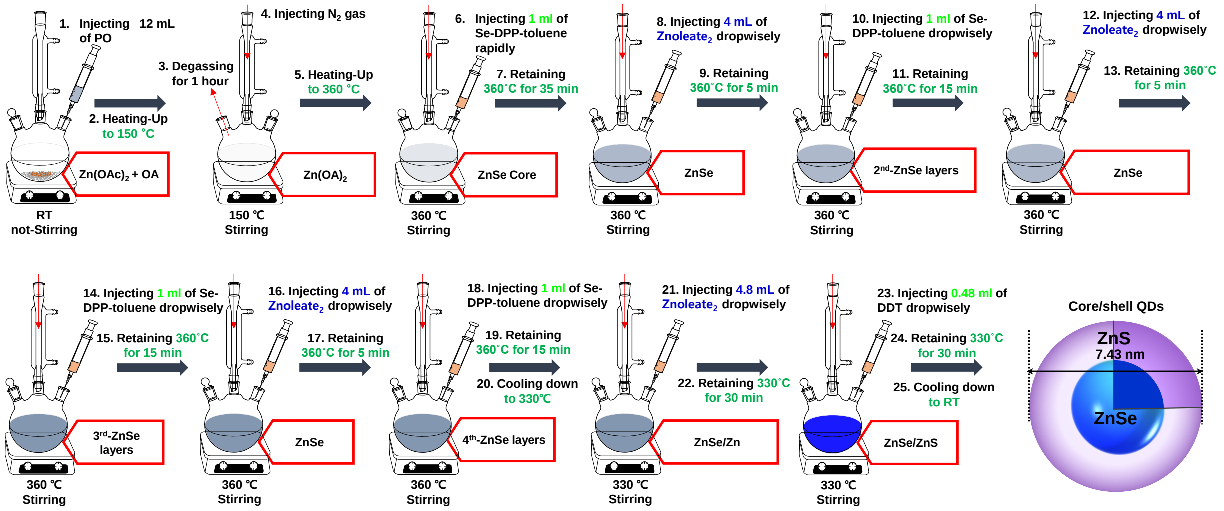

Figure S2. Schematic diagram for the synthesis process of blue-light-emitting ZnSe/ZnS core/shell QDs.

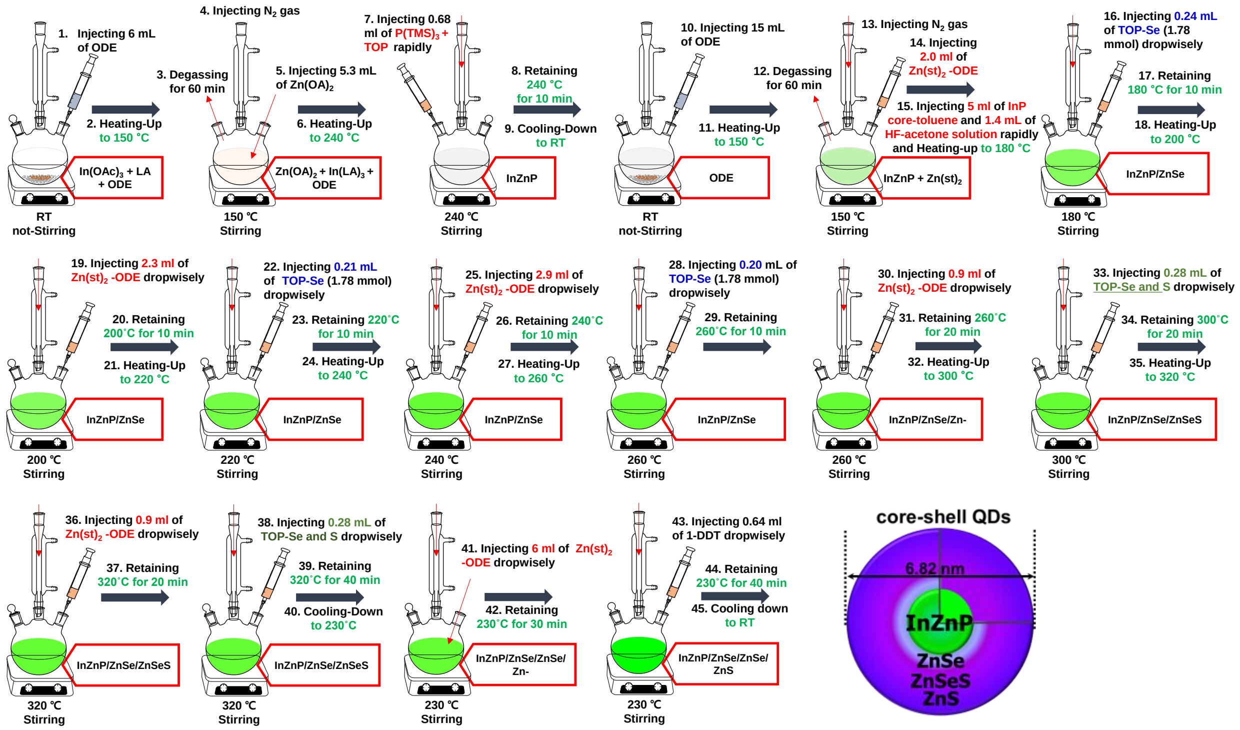

Figure S3. Schematic diagram for the synthesis process of green-light-emitting InP/ZnSe/ZnSeS/ZnS core/multi shell QDs.

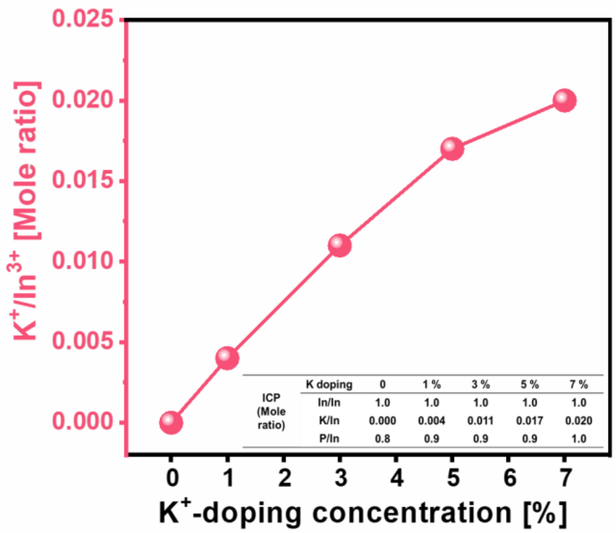

Figure S4. Quantitative analysis of atomic elements in KI-doped red-emitting In_xP_{1-x} core QDs depending on the KI doping concentration using ICP-AES.

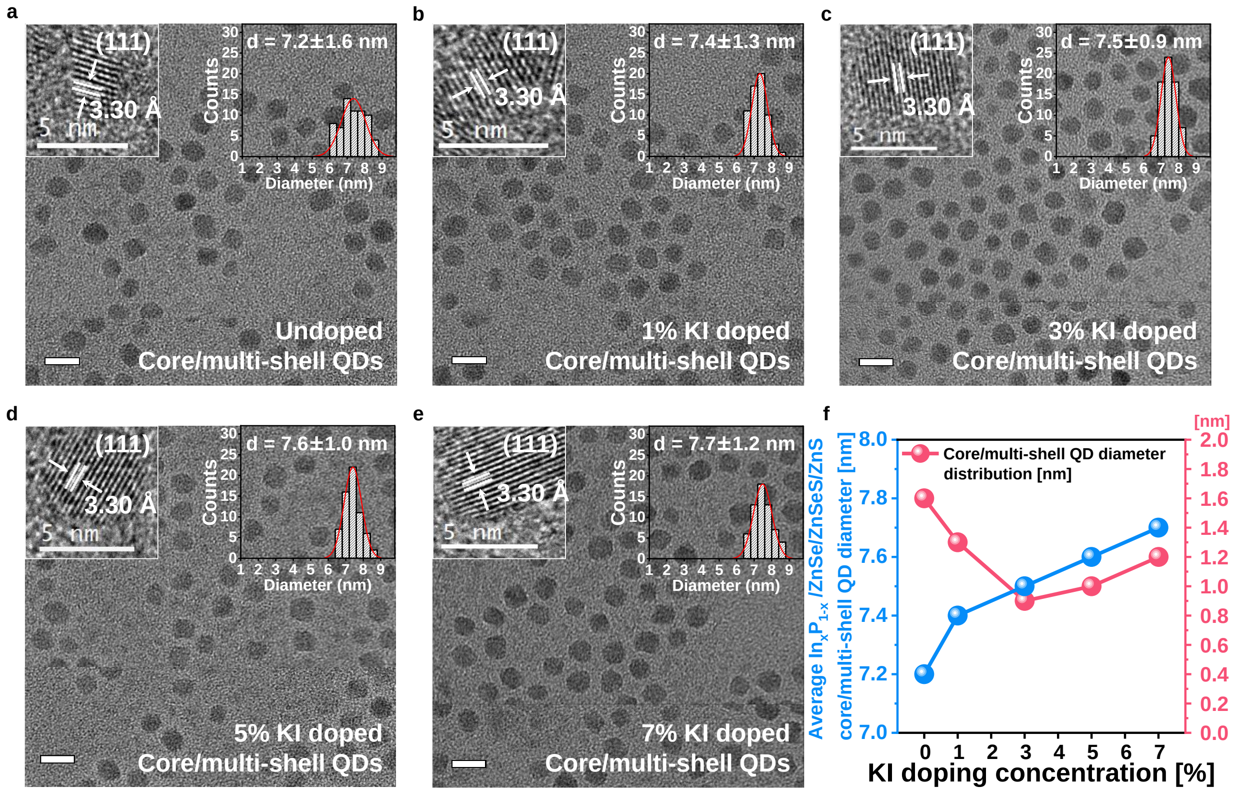

Figure S5. InP-based core/multi-shell QD average diameter and diameter distribution depending on the KI doping concentration. HR-TEM images of the In_xP_{1-x}/$Zn_{0.6}Se_{0.4}$/ $Zn_{0.6}Se_{0.1}S_{0.3}$/$Zn_{0.5}S_{0.5}$ core/multi-shell QDs for the KI doping concentration of (a) Un-doped, (b) 1 % KI, (c) 3 % KI, (d) 5 % KI, and (e) 7 % KI.

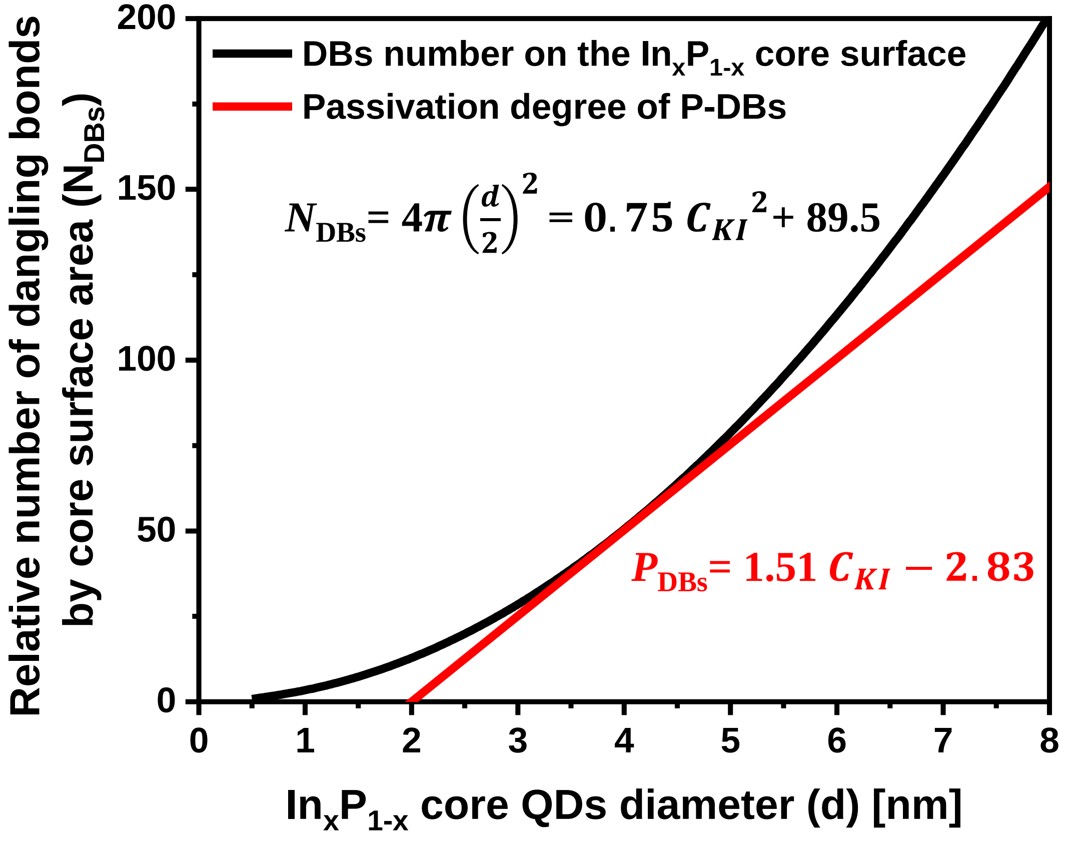

Figure S6. Dependency of the number of surface dangling bonds (N_{DBs}) versus the KI doping concentration (C_{KI}) as a function of the diameter of the In_xP_{1-x} core (d).

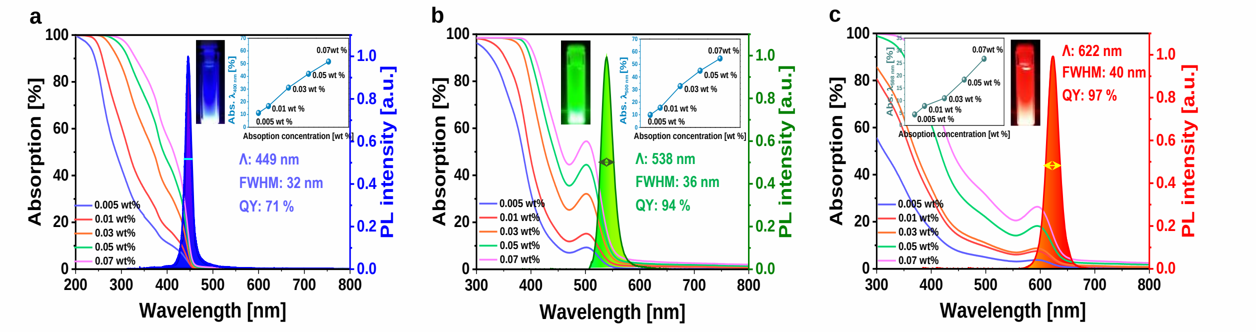

Figure S7. Optical properties of the blue-(B-), green-(G-), red-(R-)light-emitting QDs used for the QD functional-CF OLED. Absorption and PL spectra of (a) B-light-emitting ZnSe/ZnSe core/shell QDs, (b) G-light-emitting InP/ZnSe/ZnSeS/ZnS core/multi-shell QDs, and (c) R-light-emitting KI-doped $In_{0.53}P_{0.47}/Zn_{0.6}Se_{0.4}/Zn_{0.6}Se_{0.1}S_{0.3}/Zn_{0.5}S_{0.5}$ core/multi-shell QDs depending on the QDs concentrations (0.005, 0.01, 0.03, 0.05, and 0.07 wt%). Photographs (insets in a, b, and c) of solution-based QDs taken under a 365-nm handy UV lamp for B-, G-, and R-light-emitting QDs, respectively.

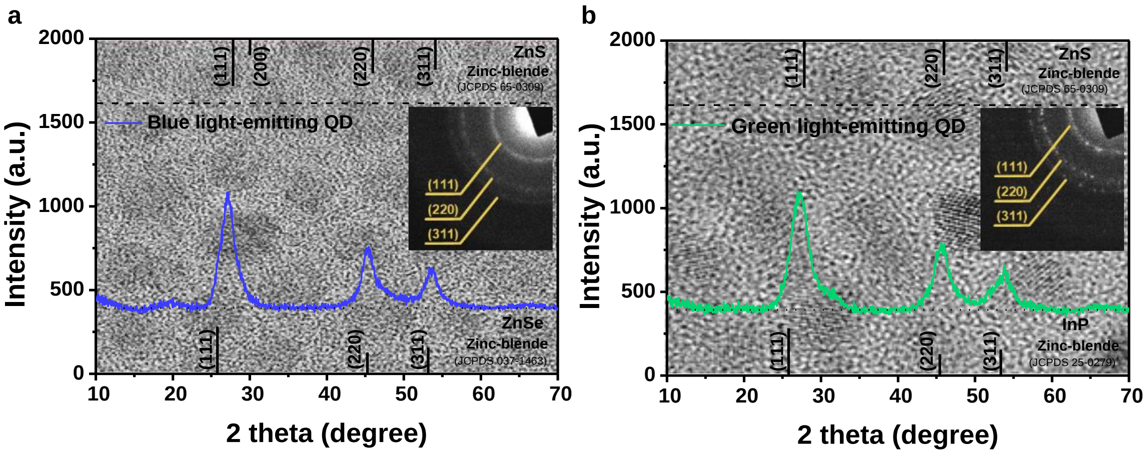

Figure S8. XRD pattern (standard JCPDS card for zinc blende ZnS, ZnSe, and InP are shown on top and bottom-line, respectively). (a) B-light-emitting ZnSe/ZnSe core/shell QDs, (b) G-light-emitting InP/ZnSe/ZnSeS/ZnS core/multi-shell QDs. Inset images show the SAED pattern.

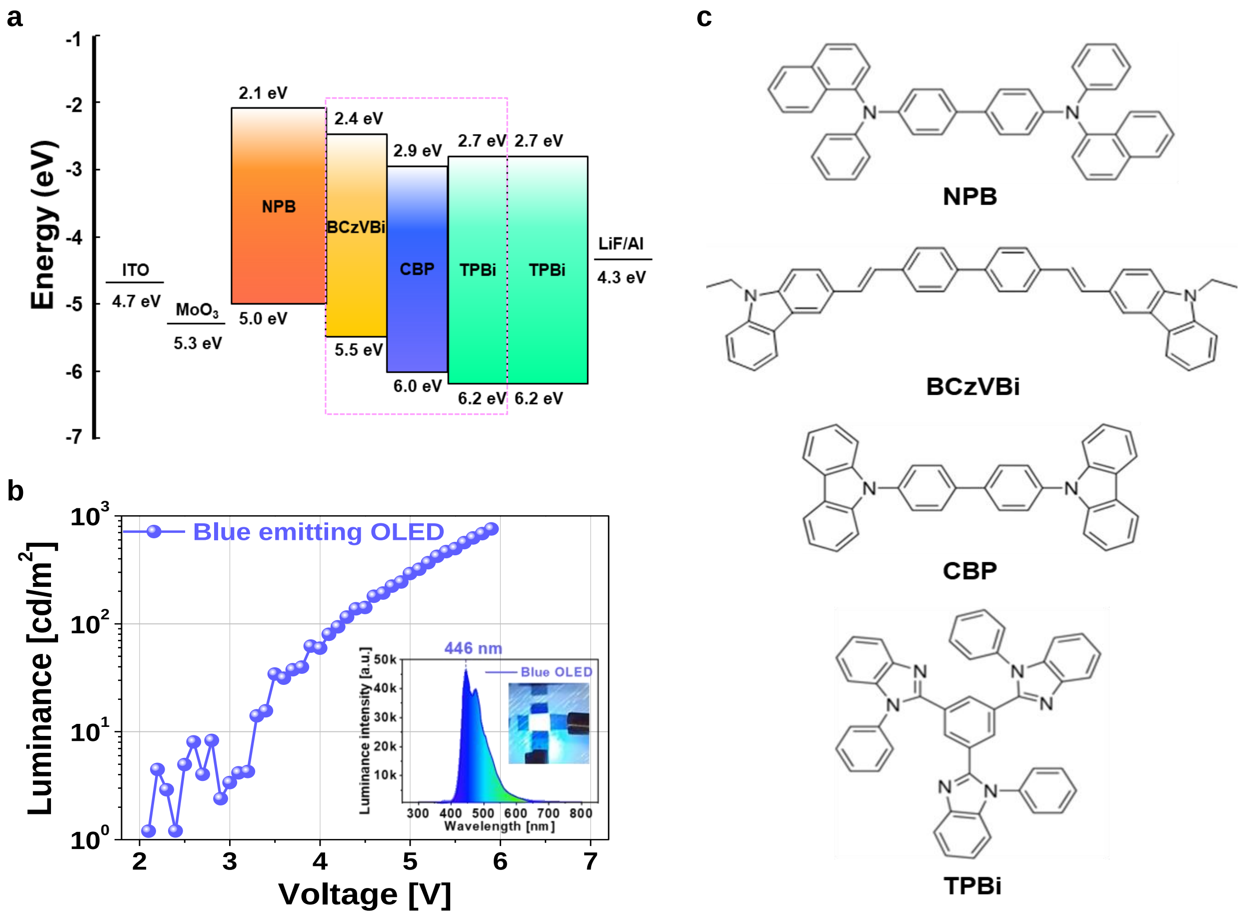

Figure S9. Optical and structural properties of blue OLED device. (a) Energy diagram of the blue OLED, (b) brightness-voltage (L-V) characteristics, inset images shows the EL spectra. (c) chemical structure of NPB, BCzVBi, CBP, and TPBi.

[S1] X. Duan, J. Ma, W. Zhang, P. Liu, H. Liu, J. Hao, K. Wang, L. Samuelson and X. W. Sun, "Study of the interfacial oxidation of InP quantum dots synthesized from tris (dimethylamino) phosphine," ACS Applied Materials & Interfaces, vol. 15, no. 1, pp. 1619-1628, 2022.
